# Development and Validation of the Short-Form BrainOK: An Efficient Digital Screening Tool for Mild Cognitive Impairment

**DOI:** 10.3390/diagnostics15243223

**Published:** 2025-12-17

**Authors:** Hyeyeoun Joo, Ye-jin Kim, Seungbo Lee, Jin-Young Min, Kyoung-Bok Min

**Affiliations:** 1 Beluga Corp, Chang-up-ro, 54, Seongnam 13449, Republic of Korea; elena.joo@belugatech.io (H.J.); isla@belugatech.io (Y.-j.K.); jake.lee@belugatech.io (S.L.); 2 Veterans Medical Research Institute, Veterans Health Service Medical Center, Seoul 05368, Republic of Korea; minymink@naver.com; 3 Department of Preventive Medicine, College of Medicine, Seoul National University, 103 Daehak-ro, Jongno-gu, Seoul 03080, Republic of Korea

**Keywords:** cognitive assessment, digitalized tool, validation

## Abstract

**Background/Objectives**: Population aging requires scalable approaches for early identification of cognitive decline, particularly mild cognitive impairment (MCI). Although the full 11-task BrainOK smartphone assessment showed excellent discrimination (AUC = 0.941), its administration time constrains large-scale use. To develop and validate a brief Short-Form BrainOK (SF-BrainOK) that preserves diagnostic performance while substantially reducing testing time. **Methods**: We enrolled 168 community-dwelling older adults (≥60 years). MCI was defined using the Montreal Cognitive Assessment (MoCA; score ≤ 23) as the reference standard. Candidate tasks were selected from the original BrainOK via LASSO-based preselection. To maximize data utilization given the limited sample size, model performance was evaluated using Leave-One-Out Cross-Validation (LOOCV). The cut-off value was determined by maximizing Youden’s J. **Results**: The final two-task model combined executive function task (Rule-based Drumming I) and memory task (Password Memory I). On the independent test set, discrimination was robust (AUC = 0.783), with sensitivity = 0.75 (95% CI: 0.63–0.85, specificity = 0.71 (95% CI: 0.62–0.80, and accuracy = 0.765 (95% CI: 0.65–0.79) at the optimal cutoff. **Conclusions**: SF-BrainOK provides a brief, two-task digital screen that markedly reduces administration time while maintaining effective diagnostic performance. By targeting executive function and memory—domains repeatedly shown to be sensitive to early MCI-related change—SF-BrainOK supports scalable, opportunistic screening and the timely identification of at-risk individuals in resource-constrained settings.

## 1. Introduction

Global population aging has fueled a sharp increase in cognitive impairment and dementia worldwide [1,2]. Currently, more than 55 million people are living with dementia, and this number is projected to nearly triple to 139 million by 2050 [1]. South Korea, one of the fastest-aging countries globally, is expected to see the number of individuals aged ≥ 65 years with dementia rise from approximately 0.83 million in 2020 to about 3.02 million by 2050 [3]. This growing burden underscores the urgent need for scalable and accessible strategies for early detection and management of cognitive decline.

Mild Cognitive Impairment (MCI), the earliest clinically recognizable stage of cognitive deterioration, has become a critical focus for early identification and intervention [4]. Early recognition of MCI allows timely therapeutic and lifestyle modifications that may delay progression to major neurocognitive disorders [5]. MCI and dementia-related conditions are no longer regarded as inevitable consequences of aging but as pressing public health priorities because of their profound impact on quality of life and the heavy economic burden they impose on healthcare and long-term care systems [6,7]. Furthermore, early detection enables the identification of potentially reversible causes, supports management of comorbidities, mitigates safety risks, and helps maintain functional independence and quality of life [8,9].

With rapid advances in digital technology and the growing need for scalable screening approaches, digital cognitive assessment tools have emerged as promising solutions for the early detection of cognitive impairment and MCI [8,9,10]. Traditional paper-and-pencil instruments, such as the Mini-Mental State Examination (MMSE) and the Montreal Cognitive Assessment (MoCA), remain valuable first-line screening tools for identifying individuals who may require further clinical evaluation for cognitive deterioration; however, their labor-intensive, in-person administration imposes substantial logistical constraints on large-scale implementation and accessibility [11]. In contrast, computerized or smartphone-based digital cognitive assessments provide a scalable and cost-efficient framework for repeated, automated, and remote testing, enabling broader reach and higher testing frequency [12]. Nonetheless, challenges related to internet connectivity, data security, and user engagement continue to limit their universal adoption [13,14,15]. However, in Korea, smartphone ownership among older adults is markedly high (56.4% in 2020 to 76.6% in 2023), suggesting that mobile-based assessments may still be highly feasible in this population [16].

Several brief screening tools, such as the Mini-Cog, have also been introduced for rapid cognitive evaluation; however, their digital implementations typically require tablet-based pencil input, which can reduce convenience in real-world settings [17]. Although limited digital familiarity among some older adults has been noted as a potential obstacle, this barrier is gradually diminishing. In South Korea—where smartphone ownership among older adults is already very high and continues to grow—a fully smartphone-native assessment may therefore offer greater practicality and real-world applicability [16].

We developed BrainOK (Beluga Inc.), a proprietary smartphone-based application for cognitive assessment [10]. The BrainOK comprises 11 distinct tasks that comprehensively evaluate multiple cognitive domains. The full version of BrainOK has been previously validated, demonstrating strong concurrent validity with the MoCA (r = 0.90, *p* < 0.001) and excellent discriminative accuracy for cognitive impairment, with an Area Under the Curve (AUC) of 0.941 (sensitivity = 0.958, specificity = 0.925) [10]. This predictive performance underscores the reliability and clinical value of BrainOK as a high-fidelity digital assessment tool. Collectively, these findings establish BrainOK as a scalable and practical complement to traditional in-person cognitive testing, facilitating earlier identification of cognitive impairment.

However, the diagnostic precision of the full 11-task version requires substantial administration time, which limits its feasibility for routine clinical use or large-scale population screening. To address this limitation, the present study aimed to develop and validate a Short-Form BrainOK (SF-BrainOK) that retains the predictive accuracy of the full version while substantially reducing testing time. We statistically optimized the original BrainOK task battery to derive a concise and psychometrically robust short form. This short form was developed to align with MoCA as the reference standard for identifying MCI. We then evaluated its reliability and diagnostic validity and established an optimized cutoff score suitable for high-volume MCI screening.

## 2. Materials and Methods

### 2.1. Study Population

We enrolled 168 older adults who visited the Seoul Veterans Health Service Medical Center between July and December 2024. Participants were recruited through convenience sampling and provided written informed consent after receiving a full explanation of the study aims and procedures.

Inclusion criteria were as follows: participants (1) aged 60 years or older; (2) who voluntarily agreed to participate after receiving a full explanation of the study purpose and procedures; and (3) who demonstrated adequate vision, hearing, and literacy to complete the cognitive assessments. Exclusion criteria included: (1) a prior clinical diagnosis of a neurodegenerative disorder (e.g., Alzheimer’s disease, Parkinson’s disease, or other dementias); (2) impaired decisional capacity related to cognitive or medical conditions; (3) severe neurological, psychiatric, or systemic illnesses (e.g., stroke, cerebral hemorrhage, or cancer) that could interfere with participation; and (4) incomplete cognitive testing, defined as failure to complete either the paper-and-pencil or smartphone-based assessment.

Cognitive function was assessed using two instruments: the MoCA and BrainOK. The MoCA was administered first, followed by BrainOK, with a minimum 30-minute interval between the two tests to minimize potential learning or fatigue effects.

The study protocol was approved by the Institutional Review Board of the Veterans Health Service Medical Center (BOHUN IRB 2024-06-013 and BOHUN IRB 2024-03-009).

### 2.2. Cognitive Assessment

#### 2.2.1. BrainOK

The BrainOK (Beluga Corp., Republic of Korea) application has been described in detail elsewhere [10]. In brief, BrainOK is a smartphone-based cognitive assessment tool developed to evaluate cognitive function in older adults using their own touchscreen devices. The software is compatible with both iOS and Android operating systems (version 0.9.25) and comprises 11 tasks covering five major cognitive domains: attention, memory, visuospatial ability, language, and executive function.

All assessments were administered in a quiet, distraction-free environment. Participants were provided with concise instructions and allowed to perform practice trials before proceeding to the main phase, requires approximately 20 min to complete.

**Figure 1:**
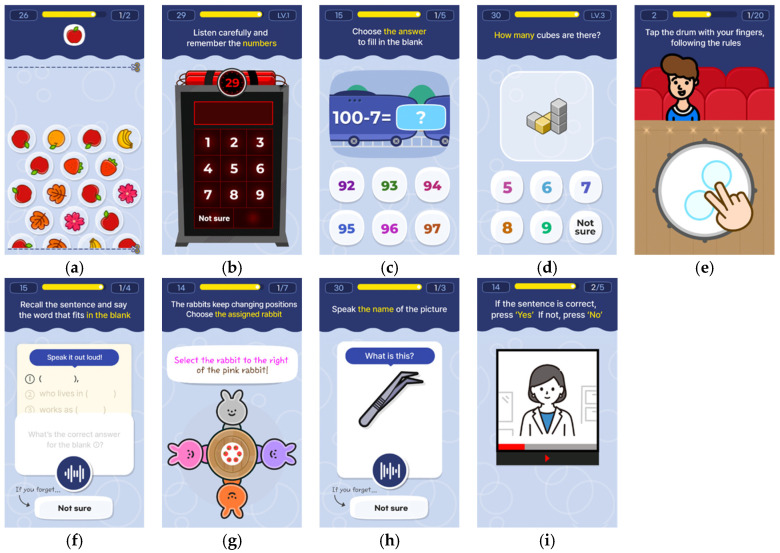
Representative screenshots of the BrainOK tasks: (**a**) Bubble cancelation; (**b**) Password memorization I, II; (**c**) Subtraction train; (**d**) Block counting; (**e**) Rule-based Drumming I, II; (**f**) Memorization of sentence; (**g**) Right–left orientation; (**h**) Naming test; (**i**) Comprehension quiz.

BrainOK provides a comprehensive evaluation across multiple cognitive domains while emphasizing accessibility, efficiency, and adaptability for older users. A detailed overview of the 11 tasks, including their corresponding cognitive domain classification and reference tasks, is presented in Table 1 and representative screenshots illustrating the interface of each task are displayed in Figure 1.

#### 2.2.2. Montreal Cognitive Assessment (MoCA)

The MoCA is a widely used brief screening instrument designed to detect MCI and early dementia with high sensitivity and specificity [29]. It is a paper-and-pencil test composed of 11 subtests that evaluate seven cognitive domains, including executive and visuospatial abilities, naming, attention, language, abstraction, memory recall, and orientation. Administration typically takes about 10 min, and scores are calculated by summing the points obtained in each domain, yielding a total of 30. In this study, a score of 23 or below was considered indicative of MCI, consistent with established diagnostic criteria. All assessments were administered and scored by trained professionals [30,31].

### 2.3. Statistical Analysis

All statistical analyses were performed using Python version 3.13 and statistical significance was set at *p* ≤ 0.05. A two-step analytical process was conducted to select candidate tasks for short-form development from the original set of 11 cognitive tasks.

First, LASSO regression analysis was applied for task preselection. LASSO is a regularized regression method that incorporates an L1 penalty (λ = 0.13), enabling automatic variable selection in addition to coefficient estimation. Therefore, it was considered an appropriate analytical method for identifying key candidate tasks.

Second, the diagnostic performance of the model was evaluated using a Leave-One-Out Cross Validation (LOOCV) strategy. LOOCV is particularly advantageous in studies with a limited sample size, as it maximizes data utilization by allowing every participant to serve as a test case exactly once, while the remaining participants are used for model training. This approach prevents data wastage and ensures that performance estimates are not overly dependent on an arbitrary train-test split. At each LOOCV iteration, one participant was held out as the test sample, and a logistic regression model was trained on the remaining data. Standardization (z-score normalization) was performed within each fold to avoid information leakage. The predicted probabilities from all test folds were aggregated to compute the Area Under the Curve (AUC), providing an unbiased estimate of generalization performance in small clinical datasets.

To determine the optimal decision threshold, probability cut-off values ranging from 0 to 1 were systematically examined, and the threshold that maximized Youden’s J index (J = sensitivity + specificity − 1) was selected. Corresponding sensitivity, specificity, and accuracy were calculated using the complete set of LOOCV predictions. Exact 95% confidence intervals were obtained using the Clopper-Pearson method.

For clinical interpretability and consistent directional comparison with MoCA scores, the LOOCV-predicted probabilities were reverse-transformed to a 0–50 scale, such that higher scores indicate better cognitive status. This transformation did not affect classification performance but enabled intuitive interpretation of cognitive severity.

## 3. Results

### 3.1. Demographic Characteristics

An overview of the demographic characteristics of the study participants, including sex, age, and years of education, is presented in Table 2.

### 3.2. Task Reduction

Five tasks were extracted by applying LASSO regression analysis. Tasks with estimated coefficients equal to zero were excluded, and only those with nonzero coefficients are presented. Table 3 summarizes the coefficients, variance inflation factors (VIF), and Pearson correlation values for the five candidate tasks. However, elevated VIF values were observed for Rule-based Drumming I (VIF = 12.373) and Rule-based Drumming II (VIF = 18.823). This was interpreted as arising from the two items’ similar characteristics and measurement of the same cognitive domain. Consequently, Rule-based Drumming II was excluded.

Following exclusion of one task from the initial preselection set, the final set of candidate predictors for SF-BrainOK model comprised the remaining four task variables—Calculator Training, Password Memory I, Sentence Memory II, and Rule-based Drumming I—together with years of education and sex. As shown in Table 4, VIFs for all predictors were below 2, indicating negligible collinearity and confirming that these predictors were sufficiently independent and stable for model estimation.

**Table 4:** Significance of Demographic Variables (Age, Sex, Education Years).

Variables	Test	*p*-Value
Age	*t*-test	0.029
Education Years	Mann–Whitney U Test	0.000
Sex	Chi-Square Test	0.913

### 3.3. Final Model Selection and Validation

For age, the normality test indicated no significant deviation from normal distribution (*p* = 0.4010); therefore, an independent *t*-test was applied, revealing a statistically significant difference (*p* = 0.0290). In contrast, education years violated the normality assumption (*p* < 0.0001), and thus the Mann–Whitney U test was used, confirming a significant group difference (*p* < 0.0001). Sex distribution was evaluated using a chi-square test, showing no significant difference between groups (*p* = 0.9135).

Based on these results, age and education were considered relevant covariates reflecting cognitive differences and were included in the subsequent modeling process, whereas sex was excluded due to its lack of association with group classification.

These analyses ensured that only meaningful demographic contributors to cognitive status were incorporated into the predictive model, while preventing unnecessary model complexity.

In Table 5, among the candidate models, the three-task combination of Rule-based Drumming I, Sentence Memory II and Calculator Train produced the highest discriminative performance. 

However, Sentence Memory II requires speech recording via a microphone, which poses practical challenges in self-administered environments. These challenges were reflected in real-world usage patterns of the original BrainOK service. Between January 2024 and December 2025, Sentence Memory II demonstrated a markedly higher dropout rate (334/2,173 users; 15.4%) than other tasks, such as Rule-based Drumming I (23/1,625 users; 1.4%) and Password Memory I (13/1,577 users; 0.8%). Given this substantially elevated attrition associated with microphone-dependent administration, Sentence Memory II was excluded on empirical grounds to enhance feasibility and user compliance in smartphone-based screening.

In contrast, Password Memory I targets the same short-term memory domain but uses touchscreen-based input, enabling more robust usability across settings and user groups. Thus, it could be served as a practical alternative to Sentence Memory II. When combined with Rule-based Drumming I and the education-years covariate, the resulting model demonstrated comparable discriminative ability while markedly improving task feasibility. Importantly, the AUC difference between the Sentence Memory II model and the Password Memory I model was only 0.003, indicating a negligible loss in discriminative performance despite the substantial gain in practicality.

Additionally, recent evidence indicates that Calculator Train, which is based on the serial seven task, shows a substantial performance gap between literate and illiterate individuals even within the non-demented group [32]. This residual education-related bias suggests a risk of overdiagnosing cognitive impairment among cognitively normal older adults with little or no formal schooling.

Moreover, adopting a two-task configuration substantially reinforces the intended role of the short-form battery as a fast, low-burden screening tool. Reducing the number of tasks directly decreases administration time and cognitive load, which is critical for large-scale or repeated assessments in community and clinical settings. The streamlined format also minimizes user fatigue and dropout risk, allowing the test to be completed more consistently across diverse populations, including older adults and digitally inexperienced users.

Therefore, despite the slightly higher AUC observed for the Sentence Memory II model, the Rule-based Drumming I and Password Memory I combination was selected as the final short-form composition.

The optimal decision threshold for classifying cognitive impairment was determined using the maximum Youden’s J Index. Among the evaluated cut-off points, a threshold score of 20 yielded the highest J value, indicating the most favorable balance between sensitivity and specificity (Table 6). Lower thresholds improved sensitivity but led to a meaningful drop in specificity, whereas higher thresholds increased specificity at the cost of poorer sensitivity.

Additionally, although age differed significantly between groups by *t*-test, its coefficient was not significant in the logistic model. Thus, age was excluded and the final SF-BrainOK classifier was constructed using two cognitive tasks (Rule-based Drumming I and Password Memory I) together with education years.

The LOOCV-derived predicted probabilities from the final SF-BrainOK model were reverse-transformed to a clinically interpretable 0–50 scale, where lower scores indicate greater cognitive impairment. As shown in Table 7, the MCI group demonstrated substantially lower SF-BrainOK scores (M = 23.88, SD = 9.78) than the normal group (M = 34.87, SD = 10.68), indicating that the short-form model successfully differentiates cognitive performance between diagnostic groups. A similar pattern was observed for MoCA, with notably lower scores in the MCI group (M = 20.12, SD = 1.58) compared to the normal group (M = 25.36, SD = 1.84), consistent with expected impairment profiles. Although the SF-BrainOK scores have relatively larger variability, group differences remained clear and directionally consistent with clinical expectations.

## 4. Discussion

This study presents the development and validation of the SF-BrainOK, which achieves a drastic reduction in assessment time while maintaining robust diagnostic fidelity. The model demonstrated good discriminative power (AUC = 0.783), which, while lower than the original 11-task BrainOK (AUC = 0.94), is considered effective for clinical screening [33,34]. The primary advantage of this statistically optimized battery is its significant efficiency over both the original BrainOK and the standard MoCA. This efficiency, combined with a high-sensitivity cutoff, indicates that the SF-BrainOK is well positioned to serve as a scalable and accessible screening tool option for identifying individuals at risk of MCI.

The final two-task composition was a pragmatic choice informed by our modeling results. The data consistently revealed that the top-performing models invariably included tasks assessing Executive Function (Rule-based Drumming I) and Memory (Sentence Memory II or Password Memory I). This finding reinforces the selection, resulting in a focused screening tool that targets the cognitive domains known to be most sensitive to early MCI-related changes. The concentration of the SF-BrainOK on these memory and executive control domains is further supported by the high efficiency of concise screening tools like the Mini-Cog, which relies on delayed recall (memory) and clock drawing (executive function) to discriminate MCI from normal aging with roughly 80% accuracy [35].

The prominence of memory and executive function in our model aligns with the canonical MCI profile, these domains are repeatedly shown to be most informative for early detection [36]. Specifically, MCI patients typically show pronounced reductions in episodic recall performance, yielding discriminative performance for distinguishing MCI from cognitively normal adults, and this aligns with prior evidence showing that most memory-based computerized tests achieve sensitivity and specificity higher than 70% and acceptable AUC values greater than 0.7 [37,38]. In addition, working-memory impairments are prevalent, as performance on the Digit Span task is significantly lower in MCI across forward (*p* = 0.029), backward (*p* = 0.020), and sequencing conditions (*p* = 0.026) [39] indicating early deficits in attention maintenance and working-memory control. Regarding executive functioning, performance on the Resistance to Interference (Contrasting Program) subtest of the FAB is significantly reduced in MCI (t = 2.44, *p* = 0.017, d = 0.56) [22], reflecting impaired inhibitory control and rule-shifting ability.

Although the present study did not directly investigate the neurobiological substrates of the SF-BrainOK tasks, prior evidence provides a conceptual framework for understanding why memory and executive function may serve as sensitive markers for early cognitive decline. The cognitive deficits observed in MCI have been associated in previous research with alterations in both the hippocampal–DMN memory system and the frontoparietal control network [40,41]. Episodic memory difficulties, for example, have been linked to disrupted coupling between the hippocampal formation and posterior cingulate cortex, as well as early tau pathology affecting medial temporal structures [42,43,44,45,46]. In addition, FDG-PET findings consistently demonstrate hypometabolism in the posterior cingulate and precuneus, supporting dysfunction within the memory network [47,48]. Similarly, executive dysfunction has been associated with changes in fronto-cingulo-striatal circuits and reduced white-matter integrity in relevant pathways [49,50,51,52]. Taken together, these multimodal findings suggest—though do not confirm for the present dataset—that memory and executive function are biologically plausible candidate domains for early detection of cognitive impairment [53,54].

This study has several important limitations. The small sample size, single-center recruitment, and convenience sampling may limit the generalizability of the findings. In particular, because of the single-center recruitment, which resulted in a sample consisting exclusively of older adults classified as either cognitively normal or as having MCI, the findings would be most appropriately interpreted within populations that share similar demographic and clinical characteristics Furthermore, participants who volunteered for smartphone-based assessment may represent a self-selected group with fewer barriers to using mobile technology compared to the general older adult population. Additionally, a crucial limitation is the reliance on MoCA cutoff scores (<23) to define cognitive impairment. While the MoCA is a widely used screening tool, this presents limitations due to its acknowledged inherent constraints, such as potential ceiling effects or cultural bias [55]. Another methodological limitation is that test–retest reliability could not be assessed due to the single-session, cross-sectional design. Future studies incorporating repeated administrations will be necessary to determine the temporal stability of SF-BrainOK performance. Critically, we did not examine direct associations between SF-BrainOK scores and pathophysiological indicators of Alzheimer’s disease pathology, such as amyloid-beta, tau, or neurodegeneration assessed via PET or MRI [56,57]. Validation against these established biological markers is necessary to confirm the SF-BrainOK’s ability to detect specific underlying pathologies and track biological disease progression. To fully establish the SF-BrainOK as a robust and clinically meaningful diagnostic tool, future validation against comprehensive, gold-standard neuropsychological batteries and both clinical diagnoses and biological markers is essential.

In conclusion, this study developed and validated the SF-BrainOK, an ultra-brief digital tool for MCI screening that demonstrated promising discriminative performance within our study population. By focusing on memory and executive function, the tool efficiently reduces assessment burden while offering practical advantages through smartphone-based self-administration. As detailed in the Limitations section, the preliminary nature of these findings—particularly the small sample size and lack of external biomarker validation—necessitates cautious interpretation. Future research with larger, diverse cohorts and biological validation will be essential to establish generalizability and clinical utility. Nonetheless, the present results suggest that the SF-BrainOK may serve as a practical and scalable approach for earlier detection of cognitive impairment.

## Figures and Tables

**Table 1:** BrainOK tasks by cognitive domain and canonical paradigms.

Domain	Test	Conventional Test
Attention	Bubble cancelation	Letter cancelation [18]
	Password memorization I, II	Digit span test [19]
	Subtraction train	Serial seven [20]
Visuospatial function	Block counting	VOSP—cube analysis [21]
Executive function	Rule-based Drumming I, II	Contrasting program, Go/No-go test [22,23]
Memory	Memorization of sentence	K-CIST [24,25]
Language	Right–left orientation	Right–left orientation [26]
	Naming test	Boston naming test [27]
	Comprehension quiz	Western aphasia battery—Yes/No Question [28]

**Table 2:** Demographic characteristics of study participants.

Variables	MCI (*n* = 65)	Normal (*n* = 103)
Female, *n* (%)	45 (67.16)	66 (64.07)
Age (year), mean (SD)	76.33 (5.2)	74.51 (5.24)
Education years < 12 y, *n* (%)	60 (89.55)	63 (61.16)

**Table 3:** Variable selection through LASSO.

Domain	Test	β	VIF	Pearson-r
Memory	Sentence Memory II	0.559	2.006	0.453
Attention	Password Memory I	0.474	4.658	0.412
Attention	Calculator Train	0.309	8.017	0.412
Executive function	Rule-based Drumming I	0.289	12.373	0.468
Executive function	Rule-based Drumming II	0.718	18.823	0.484

**Table 5:** Cross-validated discriminative performance of different task-combinations.

No. of Tasks	Model	AUC
2	Rule-based Drumming I, Sentence Memory II	0.786
3	Rule-based Drumming I, Sentence Memory II, Calculator Train	0.802
2	Rule-based Drumming I, Password Memory I	0.783
3	Rule-based Drumming I, Password Memory I, Calculator Train	0.786

**Table 6:** Diagnostic performance across thresholds (LOOCV-based predictions).

Cut Score	Sensitivity (95% CI)	Specificity (95% CI)	Accuracy (95% CI)
19	0.75 (0.63, 0.85)	0.70 (0.61, 0.79)	0.72 (0.65, 0.79)
20	0.75 (0.63, 0.85)	0.71 (0.62, 0.80)	0.73 (0.65, 0.79)
21	0.69 (0.56, 0.80)	0.75 (0.66, 0.83)	0.73 (0.65, 0.79)

**Table 7:** Comparison of SF-BrainOK and MoCA Scores by Normal vs. MCI Groups.

Group	*N*	SF-BrainOK Mean (SD) ^1^	MoCA Mean (SD)
MCI	65	23.88 (9.78)	20.12 (1.58)
Normal	103	34.87 (10.68)	25.36 (1.84)

^1^ SF-BrainOK scores are reverse-transformed to a 0–50 scale for clinical interpretability (≤20 = MCI; >20 = normal).

## Data Availability

The datasets analyzed during the current study are available from the corresponding author upon reasonable request.
